# Preventing and detecting fraud in web-based surveys with in-person recruitment: lessons for data integrity

**DOI:** 10.1186/s41073-026-00241-y

**Published:** 2026-08-03

**Authors:** April M. Ballard, Joy Monroe, Sue Sullivan, Shannon Self-Brown

**Affiliations:** 1 Department of Population Health Sciences, School of Public Health, Georgia State University, Atlanta, Georgia; 2 Center on Health and Homelessness, School of Public Health, Georgia State University, Atlanta, Georgia; 3 Single Parent Alliance and Resource Center, Atlanta, Georgia; 4 DeKalb County Housing Authority Resident Services Corporation, Decatur, Georgia; 5 Department of Health Policy and Behavioral Sciences, School of Public Health, Georgia State University, Atlanta, Georgia

**Keywords:** Online surveys, Data integrity, Duplicate participation, Participant verification, In-person recruitment

## Abstract

**Background:**

Fraud is a well-documented threat to data integrity in online research. However, little research has examined fraud risk in studies that rely on in-person recruitment but collect data online, particularly when preserving participant anonymity is necessary. The purpose of this study was to describe and evaluate fraud prevention and detection strategies in the context of an anonymous, web-based survey that relied on in-person, targeted recruitment.

**Methods:**

We evaluated fraud in a cross-sectional survey examining the experiences, needs, and barriers faced by families living in hotels in DeKalb County, Georgia. Eligibility criteria included being 18 years of age or older, currently residing in a hotel in DeKalb County, and living with at least one child under 18 years of age. Eligible participants were recruited through door-to-door outreach and received a flyer containing a survey link. Each survey entry was assessed for potential fraud using a standardized protocol comprising ten criteria and were classified as valid, potentially fraudulent, or fraudulent. Descriptive analyses were performed to summarize violations of fraud detection criteria.

**Results:**

Among 246 completed survey entries, 11 (4.5%) were initially categorized as fraudulent and 14 (5.7%) were categorized as potentially fraudulent. Duplicate IP address was the most frequently violated criterion (59.7%). However, 85.9% of these entries were ultimately classified as valid, indicating a high false-positive rate for this criterion. Other frequently violated criteria included survey duration (18.8%), time of survey submission (17.1%), and a Qualtrics duplicate flag (10.2%). Fewer than 5% of entries were flagged for email address concerns, survey response patterns, recruitment method, hotel response, demographic information, or eligibility alignment.

**Conclusions:**

These findings demonstrate that in-person recruitment does not eliminate fraud risk in online research. Fraud prevention and detection protocols should therefore be implemented proactively, even when recruitment is targeted and survey links are not publicly distributed. Rigorous approaches require advance planning, including allocating staff time for review and participant follow-up, and explicitly documenting procedures in ethics board applications and informed consent materials.

**Supplementary Information:**

The online version contains supplementary material available at https://doi.org/10.1186/s41073-026-00241-y.

## Background

Data integrity has become an increasingly significant concern in research as data collection relies more heavily on digital technologies [1–5]. Advances in Artificial Intelligence and automation have introduced new challenges for authentication, as both automated bots and human actors have become more sophisticated in their ability to evade detection and engage in fraudulent research participation [6–9]. At the same time, pressures to accelerate research production and dissemination may disincentivize or reduce the capacity for time-intensive data quality assurance practices [10]. As a result, researchers must increasingly balance the efficiencies of online data collection with the need to preserve data integrity.

Online data collection is particularly valuable for engaging hard-to-reach populations, individuals with competing demands, and members of stigmatized or marginalized groups [11–15]. It can reduce barriers to participation by allowing individuals to engage with research privately and at their own convenience, which is especially valuable when topics are sensitive or when participants may distrust institutions or researchers [1, 3, 15]. For researchers, these approaches offer logistical advantages, including lower costs, streamlined data management, reduced staffing demands, and the ability to reach participants across widespread geographic areas [1, 3, 13]. Conversely, online data collection also introduces new opportunities for fraud, which has emerged as a growing threat to research validity. Prior studies have documented multiple forms of fraudulent participation, including automated bot responses, duplicate submissions, identity misrepresentation, and inconsistent or careless responding [9, 14, 16, 17]. To address these risks, researchers have developed strategies to deter, prevent, and detect fraud, such as targeted recruitment and single-use links (deterrence), pre-survey screening and IP-based restrictions (prevention), and the review of survey metadata and personal identifiers (detection) [14, 16–21]. Notably, much of this literature focuses on studies that recruit participants through online platforms, social media advertising, or research participation portals where survey links are widely distributed or publicly accessible and fraud risks are assumed to be highest [9, 14].

Limited research has focused on the risk of fraud in studies that rely on in-person recruitment but use online data collection, particularly when preserving participant anonymity is necessary. In studies involving highly stigmatized populations, anonymity is often essential to enable recruitment, protect participants from potential harm or retaliation, and foster honest reporting [22–24]. In such contexts, traditional data integrity strategies such as identity verification, persistent identifiers (e.g., IP tracking, device fingerprints), and extensive linkage to personal information may be inappropriate or infeasible. These strategies may conflict with ethical obligations to preserve participant anonymity and confidentiality, requiring alternative approaches to ensure data integrity without introducing identifiable information [19, 25].

The current study addresses this gap by demonstrating that online surveys remain vulnerable to fraudulent participation even when recruitment occurs in person and survey links are not distributed via online platforms. The primary data integrity concern in this study was duplicate participation, whereby individuals attempt to complete the same survey multiple times to obtain compensation. This form of fraud is especially a challenge in anonymized, online research designs. Building on existing fraud prevention and detection frameworks [14, 16–21, 25], we examine how commonly used and novel strategies function and where they fall short in the context of an anonymous, online survey that relied on in-person, targeted recruitment. The purpose of this study was therefore to describe and evaluate fraud prevention and detection strategies implemented in this research setting and to highlight practical lessons that may inform future research where anonymity is essential, but data integrity must still be rigorously protected.

## Methods

### Study design

The Family Hotel Study was a cross-sectional survey that examined the experiences, needs, and barriers faced by families living in hotels in DeKalb County, Georgia. Participants were recruited from September to November 2025 through door-to-door outreach at hotels as part of a larger study estimating the number of households and individuals living in hotels in the county (described in detail elsewhere) [26]. Eligibility criteria included being 18 years of age or older, currently residing in a hotel in DeKalb County, and living with at least one child under 18 years of age. This study is reported in accordance with the 30-item Checklist for Reporting Results of Internet E-Surveys (see Supplementary Material, Appendix 1) [27].

Qualtrics [28], an online survey platform, was used to administer a web-based consent form and survey, which was pre-tested by study staff prior to launching data collection. Branching and logic were used to minimize the number of questions participants answered based on their responses, and survey questions were not randomized or alternated. The number of items per page and number of pages were dependent on the device surveys were completed on and branching and logic. The ‘back’ button was also available for respondents to change their answers, as needed.

Study staff visited 42 hotels and knocked on every door to count the number of adults and children living in each room. Eligibility was assessed in person at each hotel, after which participants received a flyer during in-person outreach that included a link to the Qualtrics survey. If no one answered the door, a flyer containing a link to an interest form for the survey was left. This form collected information on age, current hotel, household composition (i.e., number of adults and children), and contact information, allowing the study team to assess eligibility before contacting potential participants. Eligible individuals were contacted via email or text message with a link to complete the online survey, while ineligible individuals were contacted via email or text message to thank them for their time. The survey took approximately 20 min to complete, and participants received a US $25 incentive in the form of an electronic gift card upon completion.

### Fraud prevention strategies

Prior to launching the web-based survey, three strategies were implemented to deter fraud. First, the informed consent form stated that each household should not complete the survey more than once and that incentives would not be provided more than once per household. Second, participants were unable to enter their email address for compensation until the end of the survey, making the incentive process more time-consuming in an effort to discourage fraudulent submissions. Third, the Qualtrics ‘prevent indexing’ setting was enabled to block search engines from crawling or listing the survey link in search results [28], which can help reduce unsolicited or non-targeted access to the survey.

A fourth fraud prevention strategy was added after two days of pilot testing due to a higher-than-anticipated number of fraudulent entries. Specifically, an authentication field was programmed in Qualtrics that required participants to enter a unique code before accessing the consent form and survey. Codes consisted of one letter and six digits and could only be used once. Codes were uploaded using the Qualtrics contact list feature, which restricts survey access based on participant-entered credentials [28]. Individuals were locked out of the survey after three failed attempts with an incorrect or previously used code. Eligible participants received their unique code during in-person interactions at hotels, or via email or text message after completing the interest form left at unanswered hotel doors.

Notably, we intentionally did not activate the Qualtrics feature that automatically terminates a response if respondent metadata indicates the survey has already been completed [28]. Some participants did not have access to smartphones or computers and therefore completed the survey on tablets provided by the research team or on devices shared with others.

### Fraud criteria and detection strategies

Each completed survey was manually reviewed by study staff using a standardized protocol to assess potential fraud based on ten criteria (described below; Table 1), which were applied after survey completion and evaluated concurrently as part of a composite scoring approach. The full wording of survey items used to operationalize key criteria (i.e., hotel response, demographic information, eligibility consistency, and recruitment method) are provided in the Supplemental Material (Appendix 2).

**Table 1:** Fraud criteria descriptions and scoring

Criterion	Description	Point value
**System-generated metadata**		
IP address	IP address matched another completed survey entry	1
Qualtrics duplicate flag	Survey was flagged as a duplicate response by Qualtrics	1
Survey duration	Survey was completed in less than 10 min	1
Time of survey submission	Survey was completed outside of typical recruitment hours or in a batch with other entries outside of hours	1
**Response-based validation**		
Demographic information	Date of birth and at least one additional demographic characteristic (gender or relationship status) matched another entry	1
Eligibility consistency with pre-survey screening	Reported age or household composition was inconsistent with eligibility criteria assessed during pre-survey screening	3
Hotel response	Reported hotel was not visited within the prior week or was not visited during recruitment	1
Recruitment method	Participant reported an invalid recruitment method	1
Survey response pattern	Responses were internally inconsistent or ‘prefer not to answer’/‘don’t know’ was selected for five or more items	1
**Incentive/contact information**		
Email address (low concern)	Email structure suggested possible fabrication (e.g., excessive letters or numbers or misspellings)	1
Email address (moderate concern)	Email address was highly similar in structure or content to a previously compensated address	2
Email address (high concern)	Email address exactly matched that of a previously compensated participant	3

^*^All criteria were applied after survey completion and evaluated concurrently as part of a composite scoring approach. Because participant anonymity was a priority, no personally identifying information beyond contact details for incentive distribution was collected

#### IP address

The survey software automatically logged IP addresses at survey submission. IP addresses were compared across entries to identify potential duplicate participation. If an IP address matched a previously completed survey, the entry received one point. This criterion was applied cautiously, as participants using virtual private networks or devices connected through network address translation may share the same external IP address (e.g., people living in the same hotel) [29, 30], and entries completed on research team-provided tablets could generate shared IP addresses within a given hotel setting. However, IP addresses associated with cellular or mobile data are also dynamic and may change periodically, further limiting their reliability as standalone indicators of duplicate participation.

#### Qualtrics duplicate flag

The Qualtrics “prevent multiple responses” option was enabled, which places a browser cookie upon survey completion [28]. If a respondent attempted to access the survey again, the response was flagged as a duplicate. Entries flagged received one point. We did not automatically deem these entries as fraudulent because some participants completed surveys on research staff-provided tablets or shared devices.

#### Survey duration

Survey duration was assessed using Qualtrics timestamps. Surveys completed in fewer than 10 min received one point, as piloting indicated that the survey should take 15 min to complete on average.

#### Time of survey submission

Qualtrics timestamps were used to evaluate when surveys were submitted. Surveys completed outside of typical in-person recruitment hours or completed in clusters with other entries outside of recruitment hours were flagged and received one point.

#### Demographic information

Demographic information, including date of birth, gender, relationship status, and current hotel, was compared across survey entries. If date of birth and one other demographic characteristic matched those of a previously completed survey, the entry received one point.

#### Eligibility consistency with pre-survey screening

Date of birth and household composition were reviewed as a cross-check against eligibility criteria assessed during pre-survey screening. If the reported age was below 18 years old or a participant did not report living with a child under 18 years old, the entry received three points.

#### Hotel response

Participants were asked to report the hotel in which they were currently residing. If a participant selected a hotel that had not been visited in the prior week or that was not visited at any point during recruitment, the entry received one point. This point value was selected because: (1) people moved between hotels regularly, (2) hotel names were very similar and could be incorrectly reported, and (3) people shared information with friends or family living in other hotels.

#### Recruitment method

Participants were asked to indicate how they learned about the study. Response options included both valid recruitment methods used by the study team and methods not used. Selecting an invalid recruitment method resulted in one point.

#### Survey response patterns

Responses were reviewed for internal consistency and logical coherence. Surveys received one point if they contained multiple illogical responses (e.g., reporting unrealistic household size, age, and income) and/or ‘prefer not to answer’ or ‘don’t know’ was selected for five or more questions.

#### Email address

Email addresses were reviewed for potential fraud using three criteria. First, addresses consisting of alternating letters and numbers were flagged as potentially fabricated by a Bot program or a human trying to misrepresent themselves and received one point. Second, email addresses that were highly similar in structure or content (e.g., mstay92@email.com and tay92@email.com) were identified as potentially representing repeat participation and received two points. Third, if an email address exactly matched that of a previously compensated participant, the entry was automatically classified as potentially fraudulent and received three points.

### Fraud categorization

The total summed score was used to classify survey entries as valid, potentially fraudulent, and fraudulent. Point assignment and classification thresholds were determined a priori based on our prior research [18, 31] and the literature [4, 9], and were applied consistently across all survey entries. Each criterion received one point, except eligibility alignment, which received three points, and email address concerns, which received two points for moderate concern and three points for high concern. Higher point values were assigned to these criteria to reflect their relative severity and implications for data validity. Eligibility consistency was weighted more heavily because eligibility was assessed prior to providing survey access. Discrepancies therefore indicated inconsistency between screening and survey responses where participants should not have completed the survey and may have misrepresented key information. Similarly, email addresses that exactly matched those of previously compensated participants were assigned three points because they indicated a high likelihood of duplicate participation, although these entries were not automatically deemed fraudulent to allow for the possibility of shared email use, which was feasible in this context because it is possible that some of the participants did not have their own email address. Moderately similar email addresses were assigned two points to account for the possibility of distinct participants with similar contact information while still elevating concern when combined with other indicators.

Entries receiving 0–2 points were considered valid, which was based on the developed criteria and the context where we could assume that up to two fraud indicators may still reflect a legitimate response. Entries receiving three points were classified as potentially fraudulent. These participants received a follow-up email requesting confirmation of select personal information, such as date of birth, number of adults and children residing with them, and hotel they were currently residing in. Surveys receiving a score of four or more were classified as fraudulent. These participants received an email stating that their survey entry was flagged due to similarities with another submission and reiterating that only one incentive was permitted per household, as outlined in the informed consent. Messages to potentially fraudulent and fraudulent participants were designed both to deter fraudulent activity and to provide an opportunity for valid respondents to verify their submissions. Entries were classified as fraudulent and excluded from the final sample if participants failed to respond or were unable to verify their information. If participants whose entries were flagged could verify their information through follow-up communication with study staff, the entry was classified as valid and included in the final sample.

### Analyses

Descriptive analyses were performed using R Studio v.4.5.2. Frequencies and percentages were used to describe survey initiation, completion, and fraud categorization outcomes. Fraud detection criteria were examined by reporting the number and proportion of survey entries that violated each criterion across fraud classifications (valid, potentially fraudulent, and fraudulent). Additional descriptive analyses were used to characterize distributions of total fraud scores, survey completion times, recruitment pathways, and temporal patterns in survey submissions over the study period. Lastly, co-occurrence of fraud detection criteria among fraudulent and potentially fraudulent entries was descriptively assessed.

### Ethics

The institutional review board at Georgia State University approved the study protocol. All participants provided informed consent electronically before participation. A waiver of written consent was requested and approved since the study presented no more than minimal risk to participants, the survey was conducted online, and the procedures involved no activities that would normally require written consent outside of the research context.

## Results

**Fig. 1:**
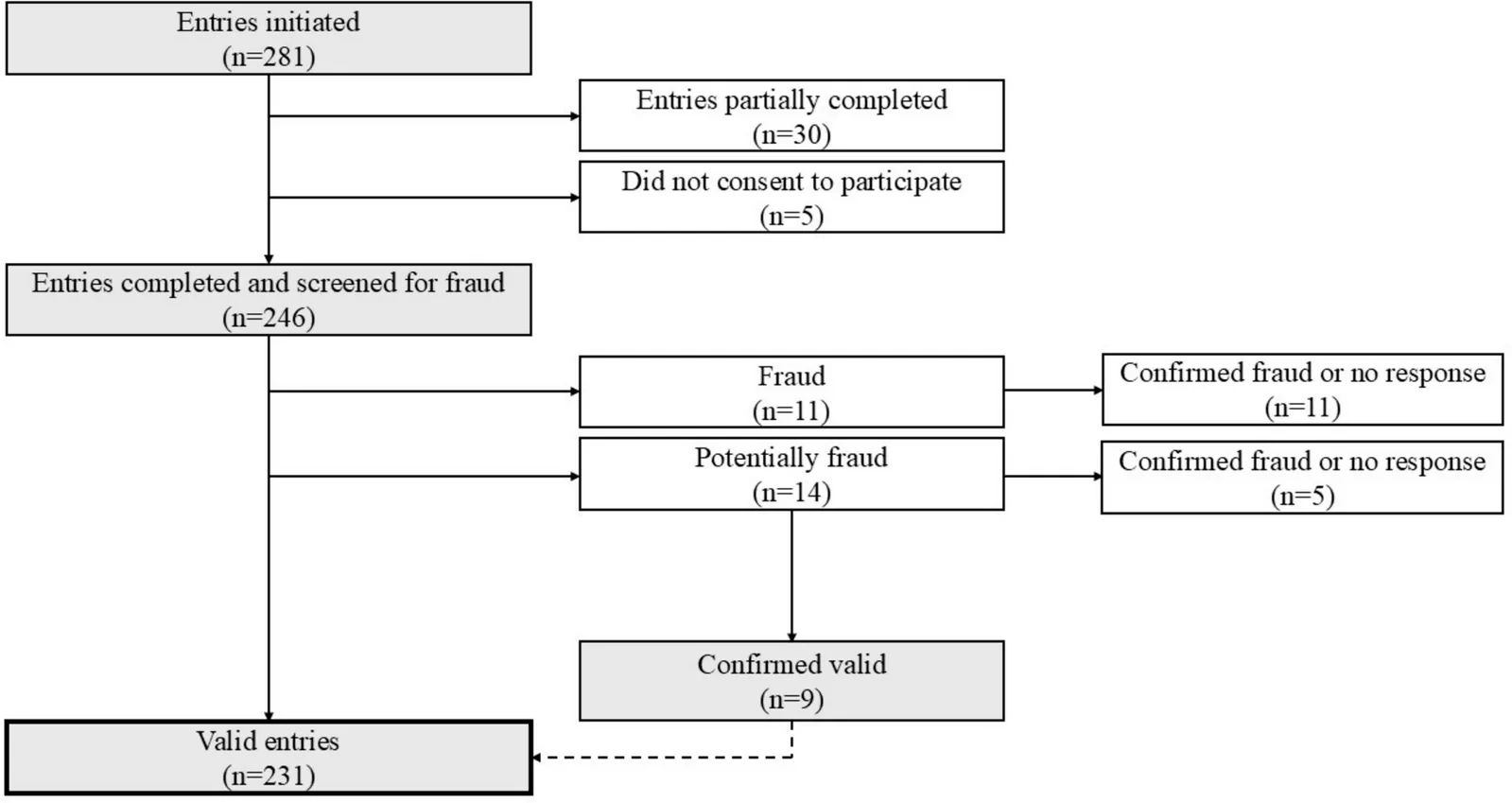
Flowchart of survey entries and fraud categorization

Figure 1 presents the distribution of survey entries by fraud classification. Overall, 281 surveys were initiated with 30 partially completed (< 50% completed) and 246 fully completed. Five participants who began the survey did not agree to participate after reviewing the informed consent form. The survey completion rate was therefore 87.5% with an average survey duration of 19 min and 19 s.

Of the 246 completed surveys, 11 (4.5%) were initially categorized as fraudulent and 14 (5.7%) were categorized as potentially fraudulent. Among these 25, 14 people responded to a follow-up email about their survey entry, nine of whom were able to verify their entries and were reclassified as valid (i.e., included in the final analytic sample). As a result, 16 (i.e., 6.5% of all completed surveys) total entries were classified as fraudulent and excluded, yielding a final analytic sample of 231 valid responses.

Fraudulent, potentially fraudulent, and valid entries were observed across both in-person and remote participant-initiated recruitment pathways. However, participants who initiated contact with the study team via the interest form, email, or text message made up a larger proportion of entries initially classified as fraudulent or potentially fraudulent than of entries classified as valid. Specifically, three of 11 (27.3%) fraudulent entries and three of 14 (21.4%) potentially fraudulent entries originated from participant-initiated contact. In contrast, only 12 of the 221 (5.4%) entries initially classified as valid originated from participants who contacted the study team remotely.

**Fig. 2:**
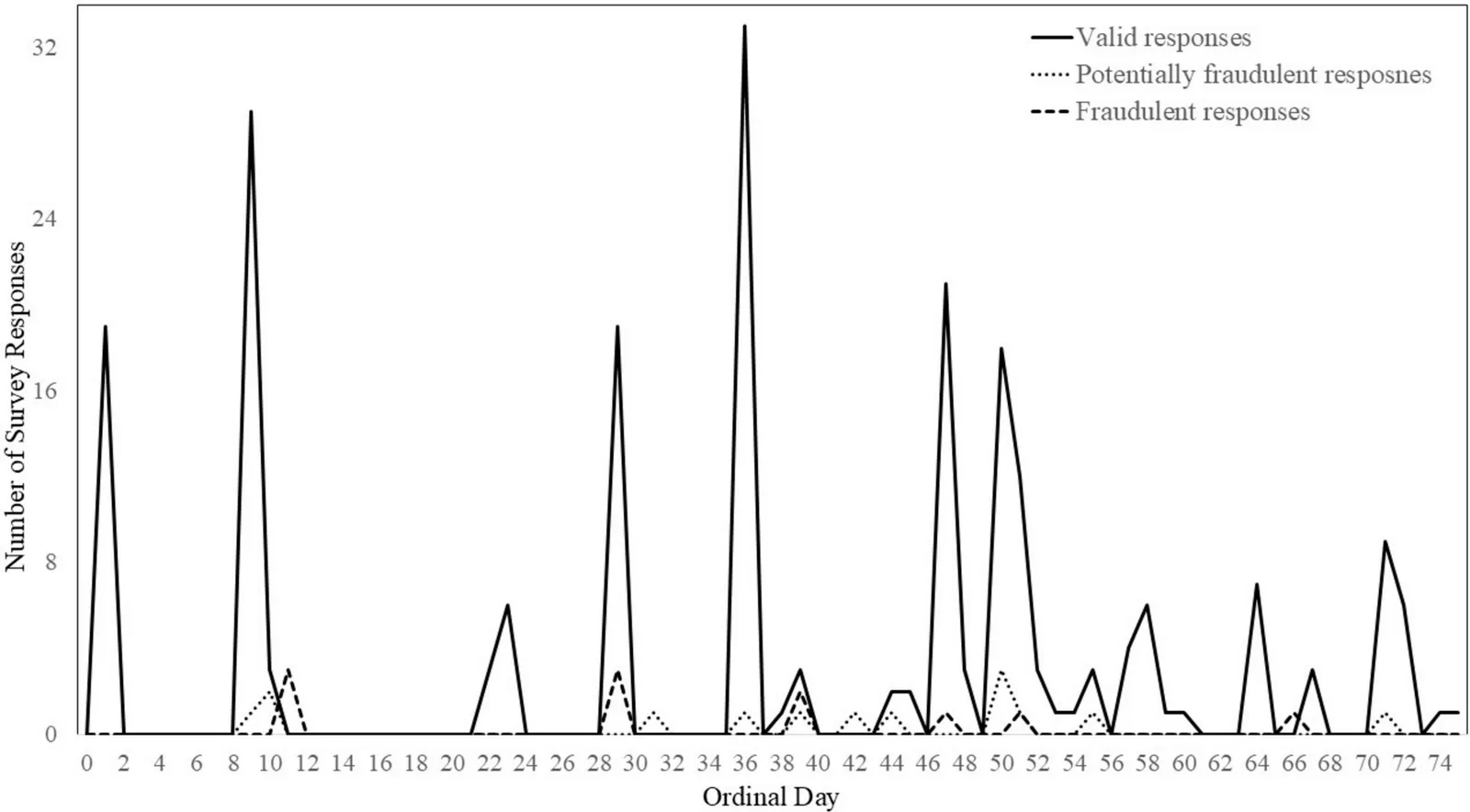
Daily change in the number of survey responses by categorization (*n* = 246)

Figure 2 shows the total number of survey entries per day throughout the study period, including the number flagged as fraudulent, potentially fraudulent, and valid per day. The early spike in fraudulent entries occurred on days 10 and 11, which were one and two days after in-person pilot recruitment. The survey was then temporarily closed and we re-opened it once we had implemented the one-time authentication codes. The introduction of the one-time codes corresponded with a marked decline in fraudulent survey submissions initially. However, the fraud detection protocol remained useful throughout the study as entries deemed fraudulent and potentially fraudulent were seen throughout the entire study period.

Surveys had the potential to receive a score of 0–14. However, the actual observed score range was 0–5. Only 38.2% (*n* = 94) of all entries received a score of zero, while approximately half (51.6%, n = 127) of entries received scores of one or two. Table 2 presents fraud detection criteria violations for the entire sample, as well as stratified by initial classification based on composite fraud scores prior to follow-up verification. The most frequently violated criterion was IP address: nearly 60% (*n* = 92) of all entries violated it. Notably, the majority (*n* = 79) of these surveys were ultimately classified as valid, indicating a high false-positive rate for this criterion. The second most frequently violated criterion was survey duration (18.8%, *n* = 39) followed by time of survey submission (17.1%, *n* = 36) and Qualtrics duplicate flag (10.2%, *n* = 25). Less than 5% of entries were flagged for email addresses (4.2%, *n* = 10), survey response patterns (3.8%, *n* = 9), recruitment method (2.9%, *n* = 7), hotel response (2.5%, *n* = 6), demographic information (1.7%, *n* = 4), and eligibility alignment (1.7%, n = 4).

**Table 2:** The number of survey entries that violated the fraud criteria across the sample and stratified by initial fraud classification prior to follow-up verification

Criterion	Total (n = 246)	Fraudulent (n = 11)	Potentially Fraudulent (n = 14)	Valid (n = 221)
	n (%)	n (%)	n (%)	n (%)
Time of survey submission	36 (17.1)	7 (63.6)	4 (28.6)	25 (11.8)
IP address	92 (59.7)	4 (36.4)	9 (64.3)	79 (35.7)
Qualtrics duplicate flag	25 (10.2)	3 (27.3)	5 (35.7)	17 (7.7)
Survey duration	39 (18.8)	4 (36.4)	6 (42.9)	29 (13.1)
Hotel response	6 (2.5)	1 (9.1)	3 (21.4)	2 (0.9)
Survey Response Patterns	9 (3.8)	4 (36.4)	3 (21.4)	2 (0.9)
Demographic information	4 (1.7)	2 (18.2)	2 (14.3)	0 (0.0)
Eligibility alignment	4 (1.7)	4 (36.4)	0 (0.0)	0 (0.0)
Recruitment method	7 (2.9)	0 (0.0)	2 (14.3)	5 (2.3)
Email address	10 (4.2)	7 (63.6)	3 (21.4)	0 (0.0)

^*^Classification reflects initial fraud categorization based on composite scoring prior to follow-up verification. Final analytic classifications differed for a subset of entries following participant verification (i.e., 9 of the potentially fraudulent entries were re-classified as valid and included in the final analytic sample)

Because the fraudulent (*n* = 11) and potentially fraudulent (*n* = 14) samples were small, it was challenging to detect definitive patterns. However, among fraudulent entries, two common pairs or combinations of violations emerged. First, three out of 11 entries (27.3%) violated time of survey submission and IP address simultaneously. Second, three of the four entries that violated eligibility alignment also violated the survey response patterns and email address criteria. Potentially fraudulent entries most commonly violated IP address, Qualtrics duplicate flag, and/or survey duration. Two out of 14 survey entries (14.3%) violated all three of these criteria, three (21.4%) violated IP address and survey duration alongside one other criterion and three (21.4%) violated IP address and Qualtrics duplicate flag alongside one other criterion. Notably, two out of the 14 potentially fraudulent entries that violated the email address criterion by using a previously paid address (high concern) did not violate any other criteria while one entry that used a similar email (moderate concern) also violated the demographic information criterion.

## Discussion

This study demonstrates that fraud prevention and detection protocols remain necessary for online data collection, even when recruitment occurs in person and survey links are not distributed through online platforms. Approximately 10% of survey entries were initially flagged as potentially fraudulent or fraudulent, and 6% were ultimately excluded from the final analytic sample. These findings suggest that fraud was a meaningful but manageable concern. In-person recruitment combined with eligibility screening reduced risk but did not eliminate opportunities for fraudulent participation, underscoring the importance of incorporating both prevention and detection strategies into study designs for online data collection in similar contexts.

The primary data integrity concern in this study was duplicate participation, whereby individuals attempt to complete the same survey multiple times to obtain compensation. Evidence of potential duplicate participation was observed across several criteria, including repeated demographic information and similar or identical email addresses. However, many flagged entries were ultimately verified as legitimate, indicating that criteria may not consistently distinguish fraudulent from legitimate participation in this context. Prior research suggests that the amount and type of compensation may influence duplicate participation, with higher incentives associated with increased risk [14, 32–35]. Although the $25 incentive offered in this study was modest, it may have created some motivation for repeat participation, particularly as multiple submissions could result in cumulative compensation. More broadly, increasing use of monetary incentives in research has heightened attention to these risks [34–38]. Notably, the study’s anonymized, online design and characteristics of the study population (e.g., shared devices, email addresses, and IP addresses due to hotel-based internet networks) complicated our ability to definitively identify duplicate participation. These findings suggest that studies involving similar populations should anticipate ambiguity in fraud criteria and incorporate verification procedures where feasible.

This study also highlights important tradeoffs between fraud prevention and detection and the protection of participant anonymity and privacy. Our protocol intentionally allowed shared devices, email addresses, and IP addresses and limited the use of identity verification and extensive linkage to personal information to support participation among a stigmatized and resource-constrained population. However, these design choices prevented us from using common techniques to address threats to data integrity (e.g., authenticating personal information such as IDs, addresses, and phone numbers) [4, 14, 35]. As a result, we used unique survey access codes to prevent fraud and a composite score and follow-up emails to detect fraud, which appeared to be effective in managing ambiguity in this context. This suggests that strategies should be tailored to the ethical and practical realities of the study population, rather than uniformly applied across settings.

The fraud classification thresholds used in this study functioned as intended, particularly an intermediate “potentially fraudulent” category. Most entries in this category were ultimately verified as legitimate through follow-up communication. This suggests that a lower cutoff for fraudulent classification would have resulted in the unnecessary exclusion of valid entries, although we did not automatically exclude any entries given that we were testing our protocol. Prior research has often used lower or more stringent thresholds [18, 20, 25], particularly in studies where personal identifying information is collected. In settings where such information cannot be ethically or feasibly obtained, adopting a higher threshold may be advantageous. These findings further highlight the value of intermediate fraud classifications that distinguish ambiguous cases from clearly invalid entries, especially in studies involving anonymity and populations where shared devices and email addresses are common. Such approaches may be particularly important when fraud is more likely to involve human actors rather than automated bots, as verification of flagged entries is more feasible in these cases. Regardless of the study context, researchers should establish fraud categories, thresholds, and follow-up procedures in advanced based on the anticipated robustness and limitations of their prevention and detection protocol [16].

Fraud prevention and fraud detection played complementary but distinct roles. The introduction of a one-time authentication code reduced fraudulent submissions and was essential to the study’s overall success. However, fraudulent and potentially fraudulent submissions continued even after this safeguard was implemented, indicating that prevention mechanisms alone were insufficient. Fraud detection, by contrast, required a comprehensive, multi-criteria approach, as half of the entries in this study violated one or two fraud criteria. Similar to prior studies, we used a combination of response-based checks, platform-generated metadata, and participant-reported information to identify potential fraud [4, 14, 35]. Our approach extends this work by integrating these indicators into a composite scoring system and combining fraud detection with preventive access controls and post hoc verification procedures. Study-specific criteria, such as recruitment method and hotel response, may also be useful to detect fraud, though these criteria were not uniquely predictive of fraud. These findings align with existing research demonstrating that no single indicator is sufficient and that combined approaches using automated checks and manual review yield the most reliable results [19, 39, 40].

Our findings suggest several practical considerations for online data collection with in-person recruitment or similar populations. First, researchers should combine access-restriction strategies (e.g., authentication codes) with post hoc detection and verification procedures. Second, fraud detection systems should incorporate multiple indicators and avoid reliance on any single criterion, particularly in contexts where shared email addresses, devices, and IP addresses are expected. Third, intermediate classifications and follow-up verification can help minimize the exclusion of valid responses. Finally, study protocols should explicitly account for the tradeoff between data integrity and participant anonymity, particularly in studies involving stigmatized or minoritized populations as prior research cautions that overly aggressive verification practices can disproportionately exclude or deter legitimate participation [5, 25, 41]. Implementing rigorous protocols requires advance planning, including allocating staff time for review, participant follow-up, and explicit documentation in Institutional Review Board applications and informed consent materials. Overall, this study highlights the need for proactive, transparent, and ethically grounded approaches to data integrity in online research, regardless of recruitment methods.

## Strengths and limitations

This study has several strengths. First, the fraud prevention and detection protocol was informed by existing research on data integrity in online studies [14, 16–21, 25] and combined well-established approaches with novel, context-specific strategies. Importantly, protocol decisions were made a priori and implemented consistently, increasing transparency and reproducibility. The study also demonstrates how methods that have been shown to be insufficient when applied in isolation [5, 25, 41], such as duplicate IP addresses or automated duplicate flags, can be meaningfully incorporated into a multi-criteria framework. Additionally, ethical considerations, including participant anonymity and allowing shared devices and email addresses, were explicitly integrated into protocol development.

This study also has limitations. Verification relied on participant follow-up via email. It is possible that some individuals provided inaccurate information during verification or did not respond, which may introduce biases or errors. Additionally, study findings may have limited generalizability to studies conducted in different contexts, as device and email sharing were reasonable and expected in this study population but may be less common elsewhere. Similarly, the use of IP address matching as a fraud indicator may have limited specificity in this context due to shared devices and dynamic IP assignment, although this indicator was interpreted cautiously and in combination with other indicators. Lastly, the potential for false negatives remains, despite the development and implementation of a rigorous fraud prevention and detection protocol. Nevertheless, the lessons learned remain broadly applicable to online studies that rely on in-person recruitment and prioritize participant anonymity while seeking to maintain data integrity.

## Conclusions

This study contributes to the existing literature by demonstrating that fraud risk remains present but manageable in studies that combine in-person recruitment with online data collection. While prior research has focused largely on studies with publicly distributed survey links or platform-based recruitment [9, 14], our findings extend this work by showing that similar challenges persist in more controlled recruitment environments. Importantly, the relatively low proportion of confirmed fraudulent entries in this study suggests that with appropriate safeguards and adjudication processes, online data collection can maintain data integrity without compromising participant accessibility or anonymity. Researchers should develop protocols in advance and remain attentive to rapid technological changes that may both introduce new vulnerabilities and improve available safeguards. Protocols must balance methodological rigor with available resources and the characteristics and needs of the study population. Approaches that are ethically grounded, context-specific, and adaptable are essential for sustaining trust, inclusion, and validity in online research.

## Supplementary Information

Supplementary Material 1.

## Data Availability

The datasets used during the current study are available from the corresponding author on reasonable request.

## References

[1] Eysenbach G, Wyatt J. Using the Internet for surveys and health research. J Med Internet Res. 2002;4(2):e862. 10.2196/jmir.4.2.e13 PMC1761932 12554560

[2] Van Gelder MM, Bretveld RW, Roeleveld N. Web-based questionnaires: the future in epidemiology? Am J Epidemiol. 2010;172(11):1292–8. 10.1093/aje/kwq291 20880962

[3] Ekman A, Litton J-E. New times, new needs; e-epidemiology. Eur J Epidemiol. 2007;22(5):285–92. 10.1007/s10654-007-9119-0 17505896

[4] Strickland IB, Ferketich AK, Tackett AP, Patterson JG, Breitborde NJ, Davis J, et al. Imposters, bots, and other threats to data integrity in online research: scoping review of the literature and recommendations for best practices. Online J Public Health Inform. 2025;17(1):e70926. 10.2196/70926 PMC12396152 40880164

[5] Simone M. Balancing data integrity concerns with ethical considerations in online research. Int J Eat Disord. 2022;55(2):290. 10.1002/eat.23674 PMC8825699 34994408

[6] See RA. Emerging threats to online security: securing systems against unauthorized automation and web bots. Staats-und Universitätsbibliothek Hamburg Carl von Ossietzky; 2025.

[7] Sakhare SR, Patil VD. Implementation of CAPTCHA mechanisms using deep learning to prevent automated bot attacks. Research Journal of Computer Systems and Engineering. 2023;4(2):01–15.

[8] Llamas JM, Vranckaert K, Preuveneers D, Joosen W. Balancing security and privacy: Web bot detection, privacy challenges, and regulatory compliance under the GDPR and AI act. Open Research Europe. 2025;5:76. 10.12688/openreseurope.19347.1 PMC11962364 40176788

[9] Pinzón N, Koundinya V, Galt RE, Dowling WOR, Baukloh M, Taku-Forchu NC, et al. AI-powered fraud and the erosion of online survey integrity: an analysis of 31 fraud detection strategies. Front Res Metr Anal. 2024;9:1432774. 10.3389/frma.2024.1432774 PMC11646990 39687573

[10] Liu J, Ma X, Jiao H, Qiu Y, Niu T, Yang B. How does academia recognize the contribution of scientific data? Evidence from data contributors’ authorship. Scientific Data. 2025;13(1):26. 10.1038/s41597-025-06340-7 PMC12800197 41331296

[11] Das M, Ester P, Kaczmirek L. Social and behavioral research and the internet: advances in applied methods and research strategies. Routledge; 2018.

[12] Schonlau M, Couper MP. Options for conducting web surveys. Stat Sci. 2017. 10.1214/16-sts597.

[13] Van Selm M, Jankowski NW. Conducting online surveys. Qual Quant. 2006;40(3):435–56.

[14] Comachio J, Poulsen A, Bamgboje-Ayodele A, Tan A, Ayre J, Raeside R, et al. Identifying and counteracting fraudulent responses in online recruitment for health research: a scoping review. BMJ evidence-based medicine. 2025;30(3):173–82. 10.1136/bmjebm-2024-113170 PMC12171401 39715631

[15] Brown R. Online interviews during a pandemic: benefits, limitations, strategies and the impact on early career researchers. PsyPag Quarterly. 2022;1(123):32–6.

[16] Lawlor J, Thomas C, Guhin AT, Kenyon K, Lerner MD, Consortium U, et al. Suspicious and fraudulent online survey participation: introducing the REAL framework. Methodol Innov. 2021;14(3):20597991211050467.

[17] Johnson MS, Adams VM, Byrne J. Addressing fraudulent responses in online surveys: Insights from a web-based participatory mapping study. People and Nature. 2024;6(1):147–64.

[18] Ballard AM, Cardwell T, Young AM. Fraud Detection Protocol for Web-Based Research Among Men Who Have Sex With Men: Development and Descriptive Evaluation. JMIR Public Health Surveill. 2019;5(1):e12344. 10.2196/12344 PMC6378547 30714944

[19] Hohn KL, Braswell AA, DeVita JM. Preventing and protecting against internet research fraud in anonymous web-based research: protocol for the development and implementation of an anonymous web-based data integrity plan. JMIR Research Protocols. 2022;11(9):e38550. 10.2196/38550 PMC9513686 36094806

[20] Sophus AI, Mitchell JW. Assessment of Fraud Deterrence and Detection Procedures Used in a Web-Based Survey Study With Adult Black Cisgender Women: Description of Lessons Learned and Recommendations. JMIR Form Res. 2025;9:e59955. 10.2196/59955 PMC11947628 40073396

[21] Pratt-Chapman M, Moses J, Arem H. Strategies for the Identification and Prevention of Survey Fraud: Data Analysis of a Web-Based Survey. JMIR Cancer. 2021;7(3):e30730. 10.2196/30730 PMC8325077 34269685

[22] Maestre JF, Eikey EV, Warner M, Yarosh S, Pater J, Jacobs M, et al., editors. Conducting research with stigmatized populations: Practices, challenges, and lessons learned. Companion of the 2018 ACM conference on computer supported cooperative work and social computing; 2018.

[23] Gabbidon K, Chenneville T. Strategies to minimize further stigmatization of communities experiencing stigma: A guide for qualitative researchers. Stigma and Health. 2021;6(1):32.

[24] Adams BL. Amplifying marginalized voices: the necessity of anonymity for diversity and inclusivity in qualitative research. Qual Res. 2025. 10.1177/14687941251350901.

[25] Liang XM, Andrews R, Hadley F, Petocz P. Online survey data integrity: lessons learnt from investigating a bot attack on a small-scale educational study. Int J Soc Res Methodol. 2026. 10.1080/13645579.2026.2636205.

[26] Monroe JS, S.; Freeman, J.; Daubon, A; DeBoer, K.R.; Self-Brown, S.; Ballard, A.M. Measuring what’s been missed: A countywide assessment of families living in extended stay hotels. Single Parent Alliance and Resource Center and GSU School of Public Health Center on Health and Homelessness; 2026. 10.57709/GSMP-AK26.

[27] Eysenbach G. Improving the quality of Web surveys: the Checklist for Reporting Results of Internet E-Surveys (CHERRIES). Gunther Eysenbach Centre for Global eHealth Innovation, Toronto, Canada; 2004. p. e34. 10.2196/jmir.6.3.e34 PMC1550605 15471760

[28] Qualtrics. Qualtrics XM Platform Provo, UT: Qualtrics; 2026 [Available from: https://www.qualtrics.com.

[29] Balakrishnan M, Mohomed I, Ramasubramanian V, editors. Where's that phone? Geolocating IP addresses on 3G networks. Proceedings of the 9th ACM SIGCOMM conference on Internet measurement; 2009.

[30] Poese I, Uhlig S, Kaafar MA, Donnet B, Gueye B. IP geolocation databases. ACM SIGCOMM Comput Commun Rev. 2011;41(2):53.

[31] Cooper HLF, Crawford ND, Haardöerfer R, Prood N, Jones-Harrell C, Ibragimov U, et al. Using web-based pin-drop maps to capture activity spaces among young adults who use drugs in rural areas: cross-sectional survey. JMIR Public Health Surveill. 2019;5(4):e13593. 10.2196/13593 PMC6913769 31628787

[32] Reips U-D. Standards for Internet-based experimenting. Exp Psychol. 2002;49(4):243. 10.1026//1618-3169.49.4.243 12455331

[33] Mustanski BS. Getting wired: exploiting the Internet for the collection of valid sexuality data. J Sex Res. 2001;38(4):292–301.

[34] Bowen AM, Daniel CM, Williams ML, Baird GL. Identifying multiple submissions in internet research: preserving data integrity. AIDS Behav. 2008;12(6):964–73. 10.1007/s10461-007-9352-2 PMC2615134 18240015

[35] Teitcher JE, Bockting WO, Bauermeister JA, Hoefer CJ, Miner MH, Klitzman RL. Detecting, preventing, and responding to “fraudsters” in internet research: ethics and tradeoffs. J Law Med Ethics. 2015;43(1):116–33. 10.1111/jlme.12200 PMC4669957 25846043

[36] Burke ANMJM. Increasing response rate in web-based/internet surveys. In: Gideon L, editor. Handbook of survey methodology for the social sciences. New York: Springer; 2012. p. 327–43.

[37] Riggle ED, Rostosky SS, Reedy CS. Online surveys for BGLT research: Issues and techniques. J Homosex. 2005;49(2):1–21. 10.1300/J082v49n02_01 16048891

[38] Shiyab W, Ferguson C, Rolls K, Halcomb E. Solutions to address low response rates in online surveys. Eur J Cardiovasc Nurs. 2023;22(4):441–4. 10.1093/eurjcn/zvad030 36827086

[39] Bonett S, Lin W, Sexton Topper P, Wolfe J, Golinkoff J, Deshpande A, et al. Assessing and improving data integrity in web-based surveys: comparison of fraud detection systems in a COVID-19 study. JMIR Form Res. 2024;8:e47091. 10.2196/47091 PMC10818231 38214962

[40] Hitches E, Dudley D, Johnstone M, Woodcock S. Bots and baddies: supporting the integrity of online survey research in the face of a growing challenge. Qual Quant. 2025;59(2):1481–506.

[41] Shaw TJ, Cascalheira CJ, Helminen EC, Brisbin CD, Jackson SD, Simone M, et al. Yes stormtrooper, these are the droids you are looking for: identifying and preliminarily evaluating bot and fraud detection strategies in online psychological research. Psychol Methods. 2025. 10.1037/met0000724. PMC12353484 40029298

